# Corrigendum

**DOI:** 10.1002/cnr2.1338

**Published:** 2021-04-08

These corrigenda serve to correct figure errors in the article by Ju et al:

In Figure 4H **,** the 40X image for the nontarget control (NTC) condition was a duplicate of the RhoB‐2 micrograph.

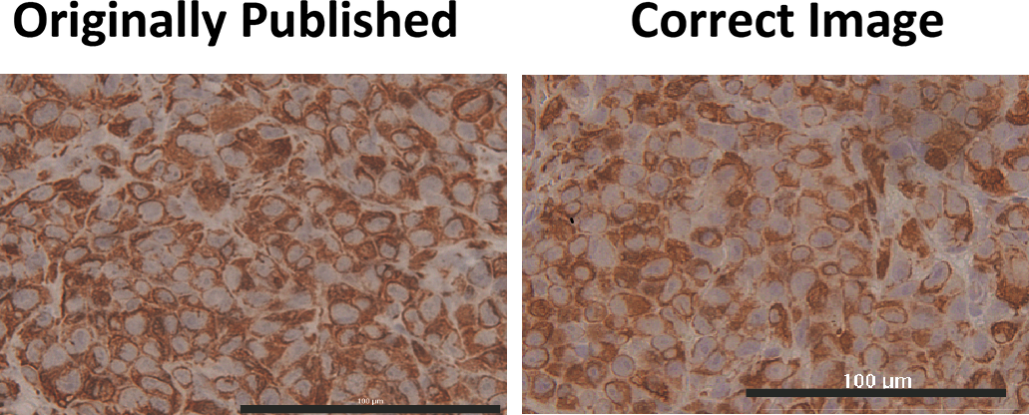

**FIGURE 4:**
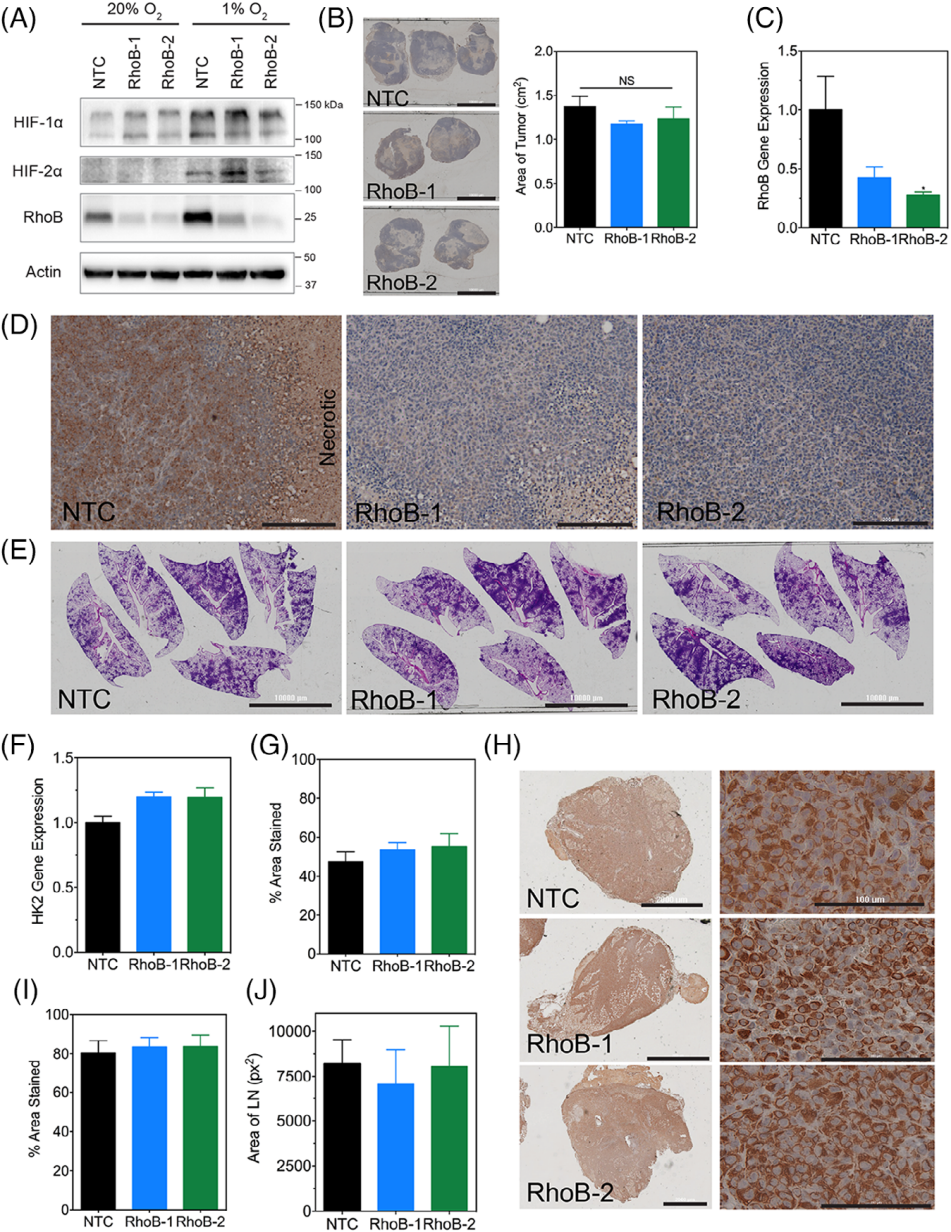
RhoB knockdown does not affect tumor growth or metastasis in vivo

The correct version of **Figure 4** is, as follows:

Additionally, in Figure 5O the overall survival (OS) curve was a duplicate of Figure 5M.

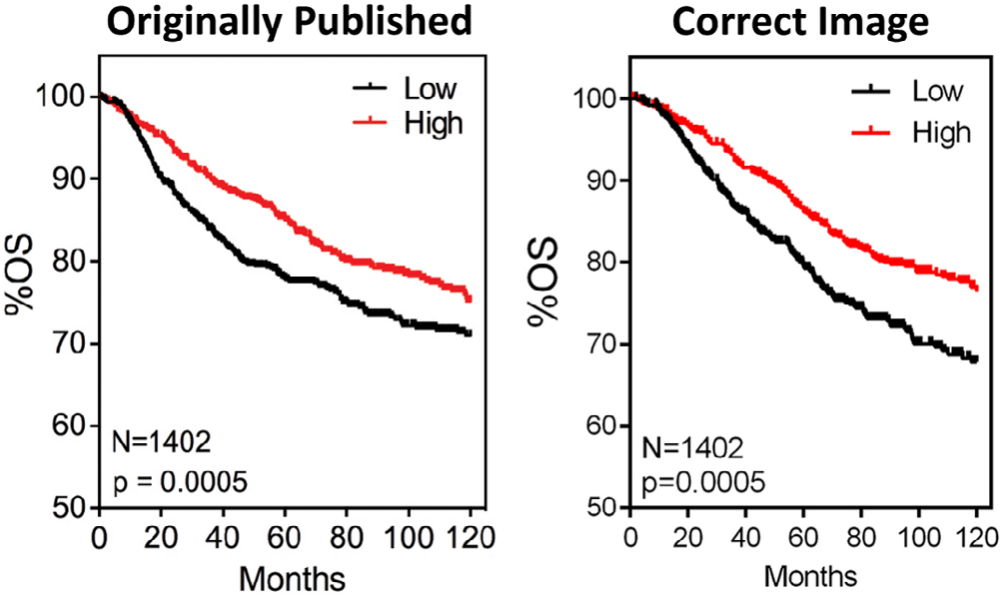

**FIGURE 5:**
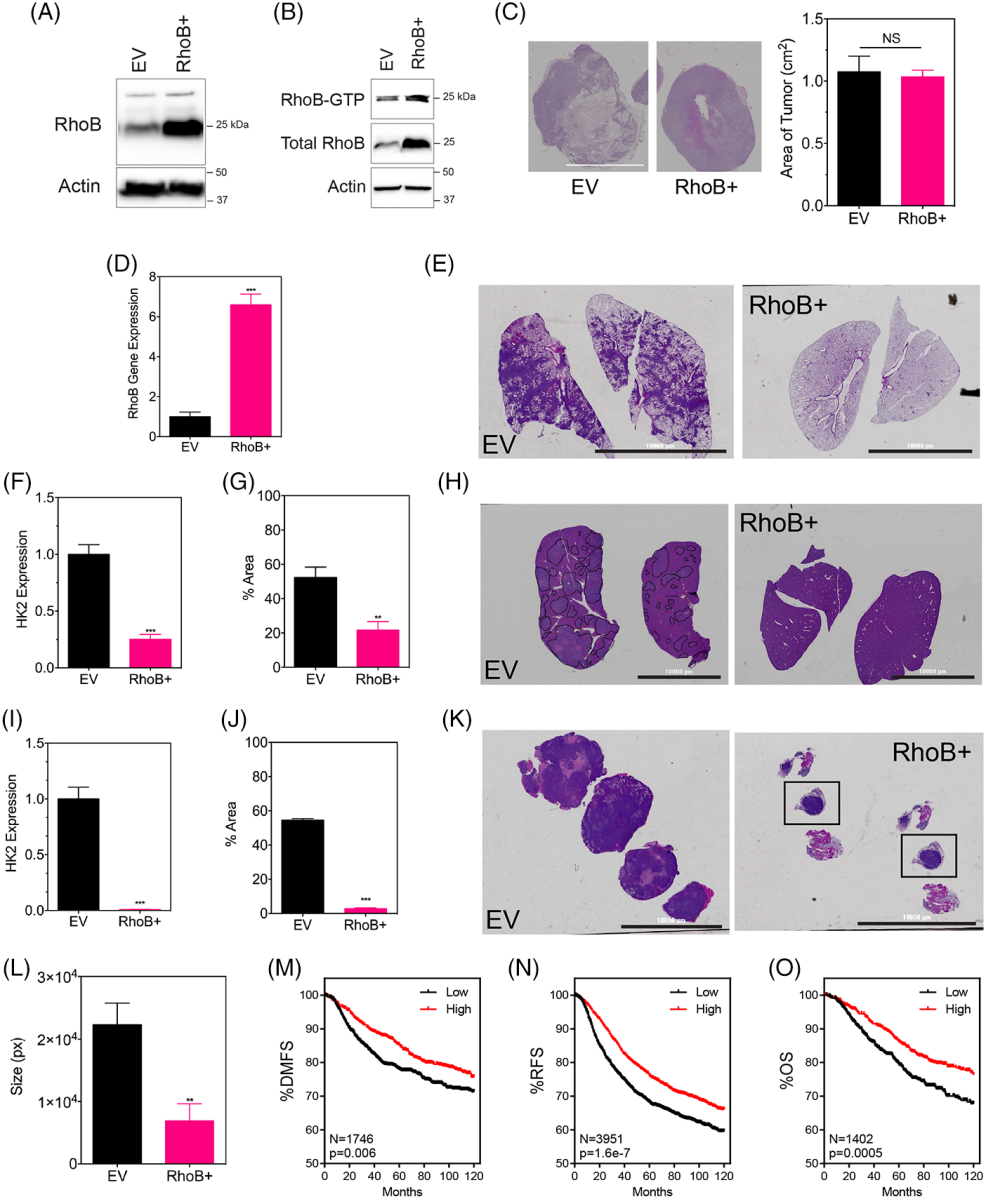
RhoB overexpression does not affect tumor growth in vivo but does contribute to a decreased metastasis

All plots were made from data using the online data portal (https://kmplot.com/) for RhoB using: Affymetrix id 212099_at, median cutoff, JetSet best probe set, and 120 months as follow up threshold.

The correct version of Figure 5 is, as follows:

Neither error alters the results or conclusions of the study. The authors regret any inconvenience this may have caused.

**Reference:**

Ju JA, Godet I, DiGiacomo JW, GilkesDM. RhoB is regulated by hypoxia and modulates metastasis inbreast cancer.Cancer Reports. 2020;3:e1164.https://doi.org/10.1002/cnr2.1164

